# Fabrication and characterization of a novel magnetic nanostructure based on pectin–cellulose hydrogel for *in vitro* hyperthermia during cancer therapy

**DOI:** 10.1039/d3ra08067f

**Published:** 2024-04-25

**Authors:** Farnoush Ahmadpour, Fatemeh Ganjali, Fateme Radinekiyan, Reza Eivazzadeh-Keihan, Milad Salimibani, Hossein Bahreinizad, Mohammad Mahdavi, Ali Maleki

**Affiliations:** a Catalysts and Organic Synthesis Research Laboratory, Department of Chemistry, Iran University of Science and Technology Tehran 16846-13114 Iran maleki@iust.ac.ir reza.tab_chemist@yahoo.com; b Department of Optics and Photonics, Wroclaw University of Science and Technology Wroclaw Poland; c Department of Industrial, Manufacturing, and Systems Engineering, Texas Tech University Lubbock TX USA; d Endocrinology and Metabolism Research Center, Endocrinology and Metabolism Clinical Sciences Institute, Tehran University of Medical Sciences Tehran Iran

## Abstract

Herein, a new magnetic nanobiocomposite based on a synthesized cross-linked pectin–cellulose hydrogel (cross-linked Pec–Cel hydrogel) substrate was designed and synthesized. The formation of the cross-linked Pec–Cel hydrogel with a calcium chloride agent and its magnetization process caused a new and efficient magnetic nanobiocomposite. Several spectral and analytical techniques, including FTIR, SEM, VSM, TGA, XRD, and EDX analyses, were performed to confirm and characterize the structural features of the magnetic cross-linked pectin–cellulose hydrogel nanobiocomposite (magnetic cross-linked Pec–Cel hydrogel nanobiocomposite). Based on SEM images, prepared Fe_3_O_4_ magnetic nanoparticles (MNPs) were uniformly dispersed in the Pec–Cel hydrogel context, representing an average particle size between 50.0 and 60.0 nm. The XRD pattern also confirms the crystallinity of the magnetic nanobiocomposite. All constituent elements and their distribution have been depicted in the EDX analysis of the magnetic nanobiocomposite. VSM curves confirmed the superparamagnetic behavior of Fe_3_O_4_ MNPs and the magnetic nanobiocomposite with a saturation magnetization of 77.31 emu g^−1^ and 48.80 emu g^−1^, respectively. The thermal stability of the nanobiocomposite was authenticated to *ca.* 800 °C based on the TGA thermogram. Apart from analyzing the structural properties of the magnetic cross-linked Pec–Cel hydrogel nanobiocomposite, different concentrations (0.5 mg mL^−1^, 1.0 mg mL^−1^, 2.0 mg mL^−1^, 5.0 mg mL^−1^, and 10.0 mg mL^−1^) of this new magnetic nanostructure were exposed to an alternating magnetic field (AMF) at different frequencies (100.0 MHz, 200.0 MHz, 300.0 MHz, and 400.0 MHz) to evaluate its capacity for an *in vitro* hyperthermia process; in addition, the highest specific absorption rate (126.0 W g^−1^) was obtained by the least magnetic nanobiocomposite concentration (0.5 mg mL^−1^).

## Abstract 2

The graphical abstract of the design and synthesis of a new magnetic nanobiocomposite based on cross-linked pectin–cellulose hydrogel substrate for *in vitro* hyperthermia of cancer therapy.

## Graphical abstract

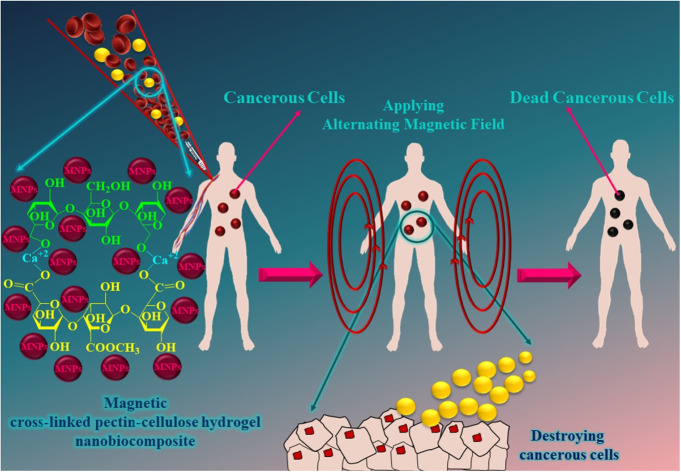

## 1. Introduction

In recent years, hydrogels possessing a specific three-dimensional network structure have been underscored because of striking characteristics: elasticity, biodegradability, biocompatibility, reversibility, and the ability to uptake high amounts of water.^1^ Hydrogels based on synthetic and natural polymers are effective substrates that render a portentous architecture employed in different scientific facets, including environmental fields,^2^ biosensors,^3^ tissue engineering,^4,5^ agriculture,^6^ and biotechnology.^7^ Compared to synthetic-based hydrogels, natural-based ones, such as agar,^8^ pectin,^9^ lignin,^10^ cellulose,^11^ chitosan,^12^ and alginate,^13^ are better candidates in green chemistry because of their biodegradable, biocompatible, and low-toxicity structure.^14^

These polydisperse polysaccharides, the most plentiful biopolymers derived from various natural resources, have specific chemical, physical, and biological characteristics.^15^ Cellulose (Cel) is the most abundant natural polysaccharide and possesses a biodegradable, biocompatible, renewable, sustainable, and eco-friendly structure with enhanced mechanical strength and low cytotoxicity. It consists of a linear polymer of β(1 → 4) linked d-glucose units.^16,17^ It is a hydrophilic substance with several hydroxyl groups that renders high inter and intramolecular hydrogen bonding and van der Waals forces and has been highlighted in biomaterial enhancement.^18^

Pectin (Pec) is a significant and naturally occurring polysaccharide known for its substantial gelling characteristics. Pec originates from plant cell walls and comprises *α*-1,4-d-galacturonic acid units.^19^ It is mostly applied as a vital gelling agent that transforms liquid samples into gels with high stability, providing favorable textures and improving the quality of products. Among other natural biopolymers, Pec individualizes itself by its outstanding gelling feature, which allows it to form gels quickly, with high thermal stability, and remarkable encapsulation capacity.^20^ Moreover, Pec is a biocompatible, non-toxic, and highly efficient natural polymer in forming hydrogels, employed in different areas, including the food industry, pharmaceuticals, drug delivery, and water treatment.^21^ For instance, Tabar Maleki *et al.* have introduced a magnetic nanocomposite based on Pec that can effectively eliminate heavy metals, such as Pb(ii), Cu(ii), and Cd(ii), from water. The nanocomposites containing metal oxides have remarkably enhanced characteristics because of the uniform dispersion of the magnetic nanoparticles (MNPs) and high aspect ratio in the polymeric context.^22^ Cross-linking between two different polymeric strings has been conducted for various hydrogels recently, *i.e.*, chitosan-Pec hydrogel,^23^ sodium alginate–carboxymethyl cellulose hydrogel,^24^ and sodium alginate–pectin hydrogel.^25^ Incorporating a cross-linker into the hydrogel helps to enhance mechanical characteristics and provide unique physicochemical responses. The cross-linking approaches are divided into physical, less stable in various conditions compared to traditional routes, and chemical, which form hydrogels with good thermal and mechanical stability.^26,27^ Due to the mild conditions of reactions, Ca^2+^ ions are the most appropriate choice for cross-linking hydrogel networks, particularly in biomedical fields. The reaction between carboxyl groups and the Ca^2+^ ions forms a hydrogel network. Recently, a Pec–Cel hydrogel has been designed and prepared using a CaCl_2_ cross-linker.^28^

Magnetic hydrogels are extensively employed for biomedical applications because of their distinctive properties. Their responsiveness and capability for remote control make these hydrogels suitable for drug delivery and hyperthermia applications. As a result, these hydrogels are promising for future studies. The thermal and mechanical characteristics of the magnetic hydrogels are tunable by altering the magnetic state, which is related to the interaction of the magnetic field and magnetic hydrogel.^29,30^ In this context, Fe_3_O_4_ MNPs have been highlighted due to their superparamagnetic behavior, large surface area, ease of surface functionalization, formation of a stable suspension, and substantial physiochemical properties.^31,32^ As documented recently by Mandal *et al.*, the Fe_3_O_4_ MNPs interact effectively with polymeric substrates *via* their functional groups. However, the surface coating of the Fe_3_O_4_ MNPs is a desirable choice to hinder their aggregation through interparticular dipole–dipole attractions.^33^ Moreover, in another work conducted by Mandal *et al.*, an Fe_3_O_4_ MNP-incorporated nanobiocomposite with improved antibacterial efficiency was synthesized through a green method and demonstrated high thermal stability and mechanical characteristics, *i.e.*, 3.31 ± 0.43 MPa tensile strength.^34^ These nanoscale agents with improved performance have been converted to ideal nano platform materials for catalytic systems,^35^ tissue engineering,^36^ sensitive magnetic resonance imaging (MRI),^37^ protein purification,^38^ and hyperthermia therapy.^39^ Apart from these descriptions, magnetic fluid hyperthermia has been considered one of the qualified nano therapy methods against cancerous cells by applying qualified Fe_3_O_4_ MNPs.^40^ Two fundamental factors, including the concertation of the MNPs and their specific absorption rate (SAR), must be considered to achieve higher efficiency in the therapeutic hyperthermia process.^41^ Considering hyperthermia treatment and applying an alternating magnetic field (AMF), these forefront NPs possessing magnetic properties and magnetic moment, oscillate and convert the magnetic energy to heat.^42^ The generated heat energy increases the intracellular local temperature of the tumor tissue, and eventually, the cancerous cells will be destroyed with the least destructive side effect on healthy tissue.^43^ To date, the surface functionalization of these qualified MNPs has been developed using a diversity of coating shells, polymers, surfactants, and targeting agents.^44^ Conversely, to reduce their toxicity, avoid the aggregation of the NPs due to their interparticle magnetic forces, and increase their biocompatibility and colloidal stability in different biological conditions, it is necessary to functionalize the surface of the Fe_3_O_4_ MNPs.^43^

**Scheme 1:**
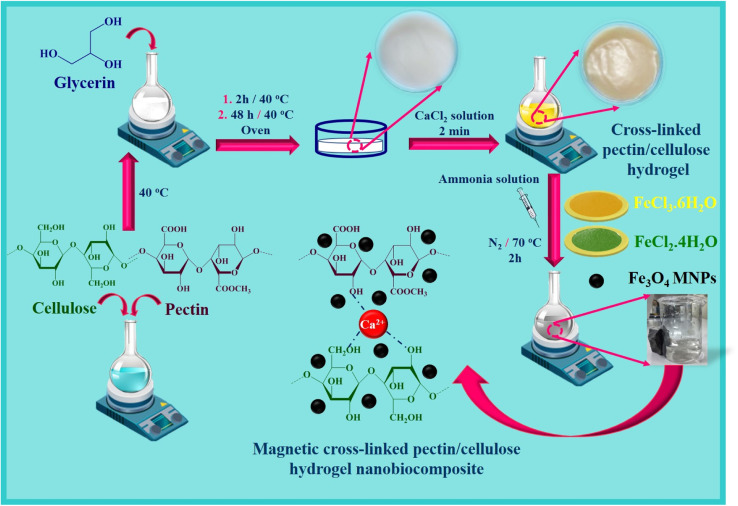
Synthesis process of the magnetic cross-linked Pec–Cel hydrogel nanobiocomposite.

In this research study, according to the unique features and multifunctionality of hydrogel structures applied in biomedicine fields, cross-linked Pec–Cel hydrogel is synthesized with a CaCl_2_ cross-linker. After that, the synthesis process of the Fe_3_O_4_ MNPs in the presence of the synthesized cross-linked hydrogel was accomplished and formed a new magnetic cross-linked Pec–Cel hydrogel nanobiocomposite (Scheme 1). This novel magnetic nanostructure was characterized by different spectral and analytical techniques, such as FTIR, SEM, VSM, TGA, XRD, and EDX analyses. In addition, to evaluate the performance and efficiency of the magnetic cross-linked Pec–Cel hydrogel nanobiocomposite for *in vitro* hyperthermia, different concentrations of this new magnetic nanostructure were examined in an AMF with different frequencies.

## 2. Experimental section

### 2.1. General

All essential chemical compounds and solvents were prepared by Merck, Fluka, and Sigma-Aldrich, international chemical companies. Several analyses were performed to characterize the structure of the magnetic cross-linked Pec–Cel hydrogel nanobiocomposite. A Fourier-transform infrared (FTIR) spectrometer (Shimadzu IR-470 model, Japan) and the KBr pellets method (200.0 to 250.0 mg of KBr powder) were used to record FTIR spectra and characterize new functional groups. Besides, considering a constant spectral resolution (4.0 cm^−1^) and defined frequency range (400 to 4000 cm^−1^), the average number of scans was conducted between 6–18. The structural elements of the designed magnetic nanobiocomposite were characterized using an energy-dispersive X-ray (EDX) spectrometer (Numerix DXP-X10P model, Czech Republic) and an ultrathin window detector. A field-emission scanning electron microscope (FESEM) (ZEISS-sigma VP model, Germany) was used to evaluate the morphology and structure of the designed magnetic cross-linked Pec–Cel hydrogel nanobiocomposite. The crystalline phase of the synthesized Fe_3_O_4_ MNPs in the structure of the designed magnetic nanocomposite was determined by X-ray diffraction (XRD) (Bruker device, D8 advance model, Germany). The vibrating-sample magnetometer (VSM) was an LBKFB model-magnetic Kashan Kavir (−10 000 Oe to 10 000 Oe) (Iran). Thermogravimetric analysis (TGA) was conducted by a Bahr-STA 504 for thermal investigations. Apart from these characterizations, considering 5 to 20 min time intervals, different concentrations of the designed magnetic nanobiocomposite (0.5 mg mL^−1^, 1.0 mg mL^−1^, 2.0 mg mL^−1^, 5.0 mg mL^−1^, and 10.0 mg mL^−1^) were exposed to an AMF at different frequencies (100.0 MHz, 200.0 MHz, 300.0 MHz, and 400.0 MHz).

### 2.2. Preparation of crossed-linked Pec–Cel hydrogel

Based on the previous literature about the preparation of hydrogels,^45^ cross-linked hydrogels based on Pec and Cel polymers were fabricated using following steps. At first, Pec (2.5 g) was dissolved, and Cel (2.5 g) was dispersed in 95.0 mL of distilled water under stirring. In the next step, glycerin solution (50.0% w/w) was added to the obtained mixer solution for 2 h at 40 °C. After that, 20.0 g of the prepared gel was cast into a Petri dish to keep for 48 h at 40 °C. The prepared gel was submerged into a solution of CaCl_2_ (0.5% w/v) and glycerin (7.0%) to prepare the crossed-linked Pec–Cel hydrogel for 2 min. After the mentioned time, the synthesized crossed-linked Pec–Cel hydrogel was kept in a Petri dish for a freeze-drying process.

### 2.3. Magnetization process of the crossed-linked Pec–Cel hydrogel as a new magnetic nanobiocomposite

To magnetize the crossed-linked Pec–Cel hydrogel, 10.0 mL of cross-linked hydrogel was weighed and mixed with 40.0 mL of distilled water. Next, 0.97 g of FeCl_3_·6H_2_O and 0.44 g of FeCl_2_·4H_2_O powders were added to the prepared suspension solution under an N_2_ atmosphere. Afterward, the mixture solution was stirred, and the temperature increased to 70 °C. Then, considering constant thermal conditions (70 °C), the ammonia solution (10.0 mL, 25.0%) was dropwise added to the mixture for 30 min. The obtained mixture solution was stirred at 70 °C for 2 h. After the mentioned time (2 h), the black precipitate was separated with an external magnet and washed with distilled water five times. Then, it was dried at room temperature.

## 3. Results and discussion

### 3.1. Preparation of the magnetic cross-linked Pec–Cel hydrogel nanobiocomposite

The essential steps have been accomplished to prepare a magnetic cross-linked Pec–Cel hydrogel nanobiocomposite. As displayed in Scheme 1, an equal amount of Pec and Cel was dissolved and dispersed in distilled water, respectively, to swell. Although the hydrogen bonding between polymeric strings is initiated, adding CaCl_2_ to provide Ca^2+^, which cross-links the polymers to form a natural polymer-based hydrogel, is crucial. The Ca^2+^ ions in the aquatic medium of the reaction contribute to forming ionic cross-links between Pec and Cel chains.^4^ Then, glycerin was added as a plasticizer to enhance the flexibility and handling properties of the hydrogel.^46^ Afterward, the *in situ* magnetization of the Pec–Cel hydrogel was carried out with iron salts, which interact with the hydrogel substrate *via* hydrogen bonding.^47^ The prepared magnetic nanobiocomposite was characterized *via* various spectroscopic and microscopic approaches, *i.e.*, FTIR, SEM, VSM, TGA, XRD, and EDX, to confirm the successful preparation of the final magnetic nanobiocomposite for *in vitro* hyperthermia applications.

### 3.2. Characterization of the magnetic cross-linked Pec–Cel hydrogel nanobiocomposite

#### 3.2.1. FTIR analysis

**Fig. 1:**
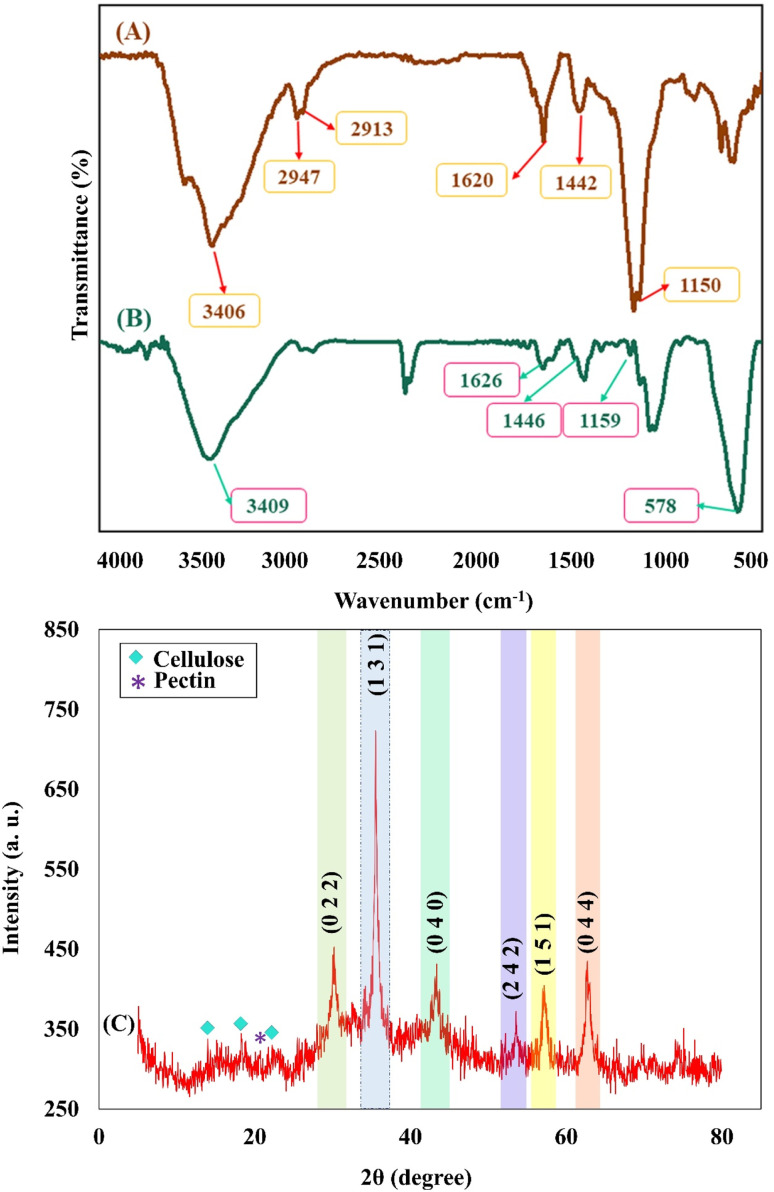
FTIR spectra of the cross-linked Pec–Cel hydrogel (A) and the magnetic cross-linked Pec–Cel hydrogel nanobiocomposite (B); XRD pattern of the magnetic cross-linked Pec–Cel hydrogel nanobiocomposite structure (C).

As can be seen in the FTIR spectrum of the cross-linked Pec–Cel hydrogel in Fig. 1A, the broad absorption band in the region of 3000–3700 cm^−1^ (3406 cm^−1^) was assigned to hydroxyl groups of Pec and Cel.^48,49^ Two absorption bands around 2947 cm^−1^ and 2913 cm^−1^ were determined for the C–H stretching vibration modes of Pec and Cel, respectively,^49,50^ and the assigned absorption band at 1620 cm^−1^ was attributed to carboxyl groups.^51^ The presence of –CH_2_ groups of the Pec structure was determined by observing an absorption band at around 1442 cm^−1^.^52^ The absorption band at 1157 cm^−1^ was attributed to the C–O stretching vibration mode of the polymer.^53^ Apart from mentioned absorption bands in the FTIR spectrum of the magnetic cross-linked Pec–Cel hydrogel nanobiocomposite (Fig. 1B), a strong absorption band that was observed at around 578 cm^−1^ was related to the Fe_3_O_4_ MNPs in the structure of the designed magnetic nanobiocomposite,^54^ indicating a prosperous magnetization of the nanobiocomposite *via* creating a network of hydrogen bonding. Another possible interaction is coordinating the hydrogels' hydroxyl groups to the unoccupied orbitals of Fe in Fe_3_O_4_.^55^

#### 3.2.2. XRD pattern

The XRD pattern of the magnetic cross-linked Pec–Cel hydrogel nanobiocomposite is conducted from 5° to 80° and indicated in Fig. 1C to evaluate the crystallinity of the prepared nanobiocomposite. As can be seen, the diffraction peaks of the Fe_3_O_4_ MNPs can be detected in the XRD pattern of the final nanobiocomposite, exhibiting its successful magnetization (Fig. 1C). The crystalline peaks at various diffraction angles (2*θ* = 30.16°, 35.53°, 43.17°, 53.57°, 57.10°, and 62.71°) comply with the standard XRD pattern of Fe_3_O_4_ MNPs (JCPDS card no. 96-900-5839).^56^ The observed crystalline peaks were characterized by their indices ((0 2 2), (1 3 1), (0 4 0), (2 4 2), (1 5 1), and (0 4 4)). Besides, the XRD pattern of Pec demonstrates an amorphous structure with the characteristic peak arising at 2*θ* = 20.0°.^57^ Moreover, the diffraction peaks at 2*θ* = 14.8°, 16.2°, and 22.7° confirm the crystalline structure of the Cel.^58^ The crystallinity percentage of the Cel has been calculated *via* various methods, such as FTIR and XRD.^59,60^ According to this, the crystallinity of the Cel in the magnetic cross-linked Pec–Cel hydrogel nanobiocomposite has been computed *via* the following eqn (1). The Cel's crystallinity was calculated to be ∼3.0%.1
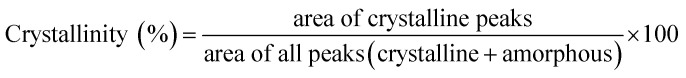


#### 3.2.3. FESEM imaging

**Fig. 2:**
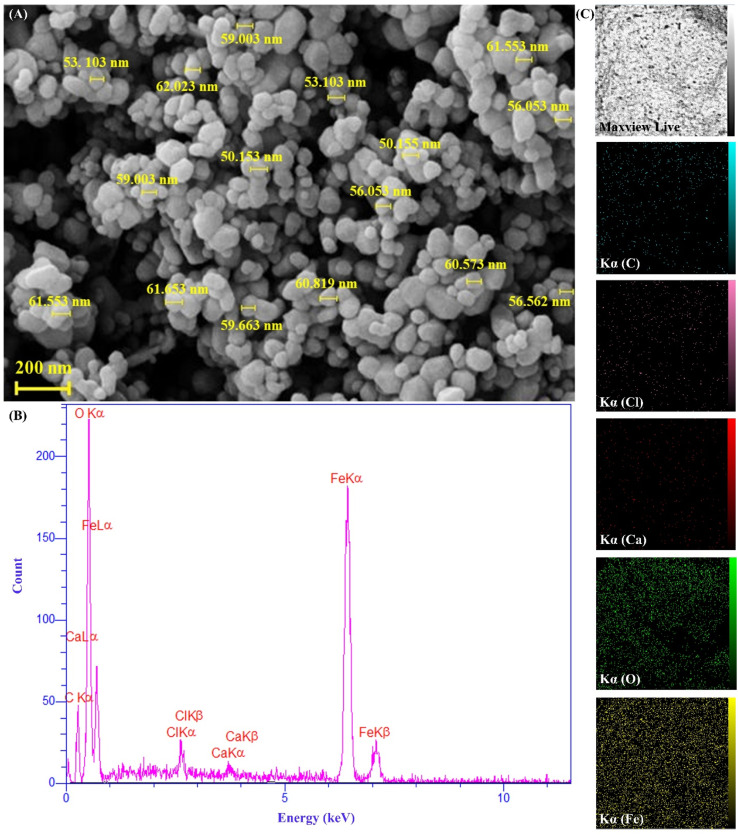
FESEM image (A), EDX spectrum (B), and elemental mapping images of the magnetic cross-linked Pec–Cel hydrogel nanobiocomposite (C).

As can be seen, the FESEM image of the magnetic cross-linked Pec–Cel hydrogel nanobiocomposite is indicated in Fig. 2A. Considering the FESEM imaging results, it can be mentioned that the *in situ* preparation of Fe_3_O_4_ MNPs in the presence of cross-linked Pec–Cel hydrogel has been well conducted, and these MNPs with an almost spherical morphology and a unique distribution have covered the structure of the hydrogel substrate. In addition to this, the average diameter of the synthesized Fe_3_O_4_ MNPs was estimated to be between 50.0 and 60.0 nm.

#### 3.2.4. EDX analysis

As illustrated in the EDX spectrum of the magnetic cross-linked Pec–Cel hydrogel nanobiocomposite (Fig. 2B), calcium and chlorine peaks can be attributed to the cross-linked calcium chloride agent. Carbon and oxygen peaks confirmed the presence of Pec and Cel biopolymers. Besides, oxygen and carbon peaks can be related to Fe_3_O_4_ MNPs. Considering the elemental peaks observed in the spectrum, their distribution pattern was determined by elemental mapping images (Fig. 2C).

#### 3.2.5. VSM analysis

**Fig. 3:**
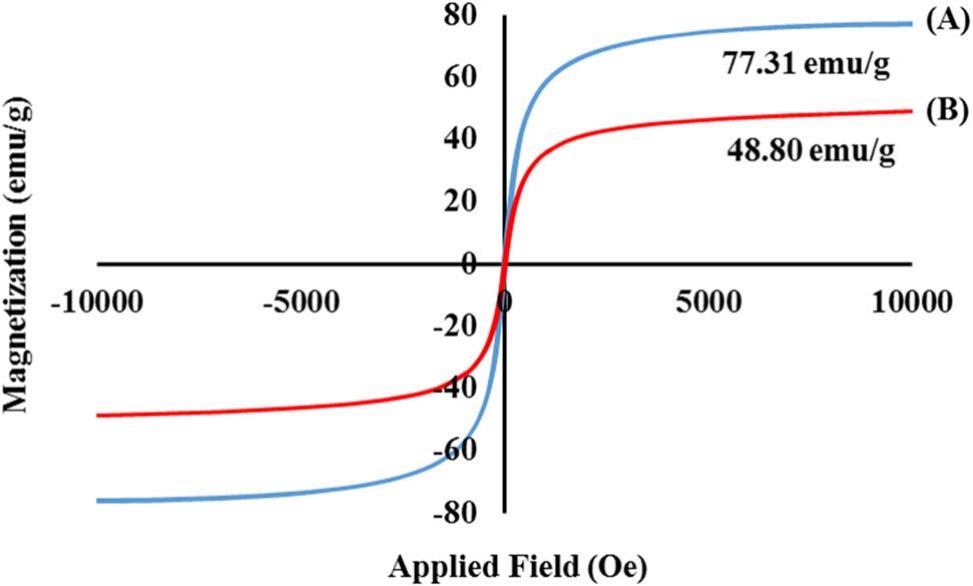
Hysteresis loop curves of the bare Fe_3_O_4_ MNPs (A) and magnetic cross-linked Pec–Cel hydrogel nanobiocomposite (B).

Considering a magnetic field between −10 < kOe < +10, the magnetic properties of the magnetic cross-linked Pec–Cel hydrogel nanobiocomposite were evaluated by a VSM instrument. Several important factors, such as the size of the MNPs, shell thickness, number of shells, and overall nature of the MNPs, can impact the saturation magnetization value of the MNPs.^61,62^ In addition, the difference in saturation magnetization value of the MNPs can be ascribed to the temperature and the method used for their synthesis process. As indicated in Fig. 3A and B, the saturation magnetization value of the bare Fe_3_O_4_ MNPs without any further functionalization was compared with the magnetic cross-linked Pec–Cel hydrogel nanobiocomposite. In comparison to the Fe_3_O_4_ MNPs with a saturation magnetization value of 77.31 emu g^−1^, a considerable decrease in saturation magnetization value (48.80 emu g^−1^) of the designed magnetic nanobiocomposite can be related to the formation of a core–shell structure and covering of the synthesized Fe_3_O_4_ MNPs by Pec and Cel polymeric chains.

#### 3.2.6. Thermal analysis

**Fig. 4:**
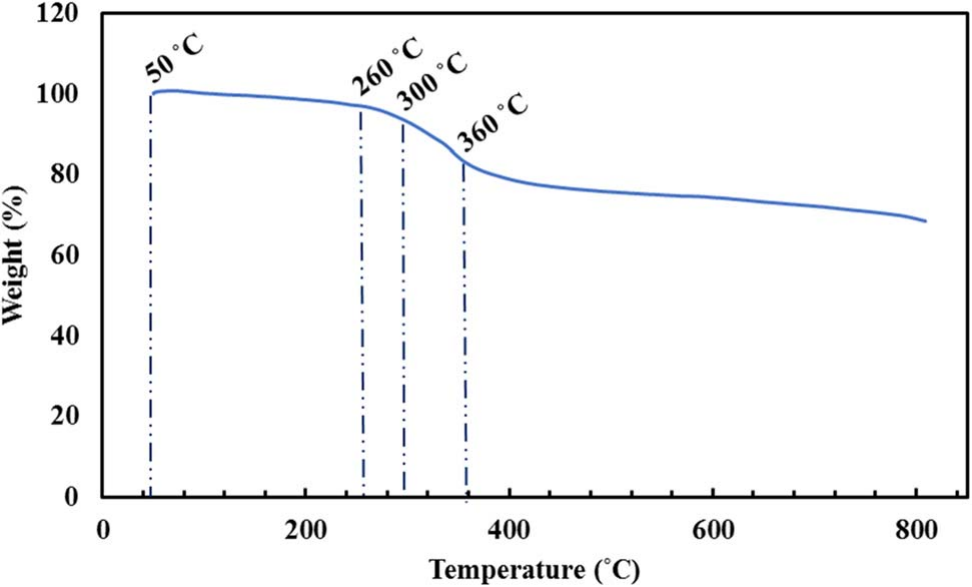
TGA curve of the magnetic cross-linked Pec–Cel hydrogel nanobiocomposite.

Thermogravimetric analysis (TGA) was applied to examine the nanocomposite components' thermal stability. Fig. 4 demonstrates the TGA of the magnetic cross-linked Pec–Cel hydrogel nanobiocomposite. The magnetic nanocomposite's mass decrease was evaluated in the approximately 50–800 °C temperature range. The applied atmosphere and steady heating rate were argon and 10 °C min^−1^, respectively. The initial mass loss (4.0%) was observed at 50–−260 °C associated with the loss of trapped water and solvents. Furthermore, by elevating the temperature from 260 °C to 360 °C, the main weight loss of *ca.* 14.0% occurred assigned to the hydrogel organic constituent thermal dissociation. For instance, the Pec hydrogel degradation is in this temperature range.^23^ The Cel backbone decomposition initiates at 300 °C and proceeds to 800 °C with a meager slope.^63^ The residual weight at 800 °C was about 68.6%, attributed to the remaining ash and Fe_3_O_4_ MNPs.

## 4. Hyperthermia application of the magnetic cross-linked Pec–Cel hydrogel nanobiocomposite

**Fig. 5:**
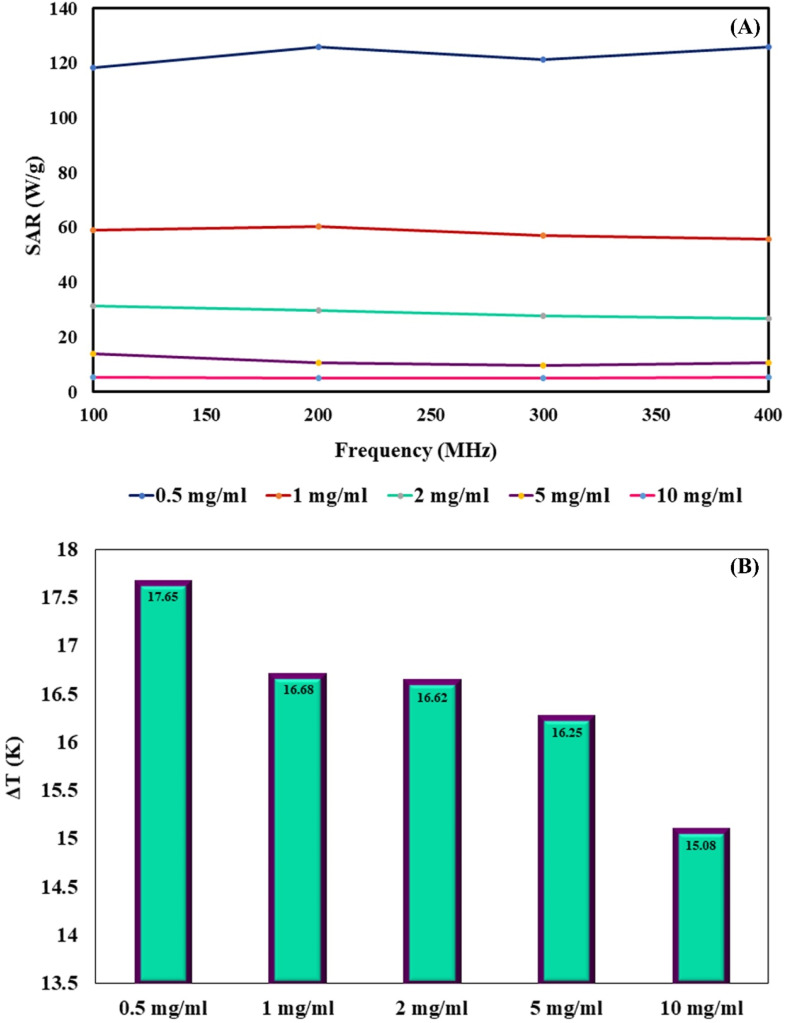
Variation of SAR with AMF frequency for each magnetic cross-linked Pec–Cel hydrogel nanobiocomposite concentration (A). Maximum Δ*T* obtained by each concentration of magnetic cross-linked Pec–Cel hydrogel nanobiocomposite (B).

MNPs have certain advantages over bulk materials in bioscience. Namely, due to their small size, they can penetrate various biological materials, and their high surface-to-volume ratio makes them very reactive. When MNPs are placed in an AMF, they release energy in the form of heat. The amount of this energy depends on the properties of the NPs and applied magnetic field. This effect has been recently used for hyperthermia in cancer treatment applications. The heating capabilities of the magnetic cross-linked Pec–Cel hydrogel nanobiocomposite were measured in this research study. To prepare a solution with a certain concentration of designed magnetic nanobiocomposite, it was placed under an AMF with a certain frequency for 20 min, and an increase in the temperature was measured by a thermocouple in five-min intervals. This experiment was repeated for MNP samples with 0.5, 1.0, 2.0, 5.0, and 10.0 mg mL^−1^ concentration and AMF frequencies of 100.0, 200.0, 300.0, and 400.0 MHz for a total of twenty tests. The specific absorption rate (SAR) was calculated using the following eqn (2) to evaluate the thermal performance of the designed magnetic nanobiocomposite.2
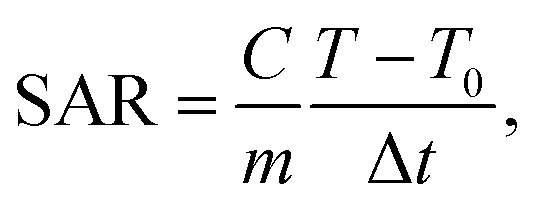
where *C* is the specific heating capacity of the sample, *m* is the concentration of magnetic nanobiocomposite in the sample, *T* is the temperature of the sample at any given time, *T*_0_ is the initial temperature of the MNPs, and Δ*t* is the time duration. Each sample was kept at the temperature of 25 °C before any test. Fig. 5A illustrates the variation of SAR with AMF frequency for each sample. As expected, the highest SAR was achieved by the sample with the least magnetic nanobiocomposite concentration, as the dipole–dipole effect between the NPs can interfere with their heat generation. Furthermore, these results show a significant drop in SAR by an increase in the sample concentration from 0.5 mg mL^−1^ to 10.0 mg mL^−1^, where SAR is reduced from 126.0 W g^−1^, obtained from 0.5 mg mL^−1^, to about 5.0 W g^−1^ in the 10.0 mg mL^−1^ sample. While the effect of the sample concentration on the SAR is quite drastic, the impact of the AMF frequency is much less significant. While the SAR lowers by more than 95.0% from the lowest concentration sample to the highest, the variation of SAR by the AMF frequency is 6, 7, 14, 30, and 2 percent for the 0.5, 1.0, 2.0, 5.0, and 10.0 mg mL^−1^ samples. This shows that the effect of the AMF frequency variation varies for different concentration samples. While these results show that by lowering the magnetic nanobiocomposite concentration in the sample, we can reach higher thermal power per unit of mass, it is essential to compare the pure temperature difference generation obtained from each sample to evaluate their performance better.

Fig. 5B shows the maximum Δ*T* caused by each sample. This chart shows that an increase in the sample concentration reduces the SAR and final Δ*T* achieved by them. Overall, the wide range of thermal powers that the designed magnetic nanobiocomposite can achieve makes it a very versatile tool for many hyperthermia applications in the biomedical field.

## 5. Conclusions

In summary, the synthesis process of the cross-linked Pec–Cel hydrogel substrate using natural Cel and Pec biopolymers and CaCl_2_ cross-linking agent and *in situ* preparation of Fe_3_O_4_ MNPs in the presence of this natural-based hydrogel were accompanied by the formation of a new magnetic and heterogenous nanobiocomposite. The structural features of the magnetic cross-linked Pec–Cel hydrogel nanobiocomposite were evaluated by FTIR, SEM, VSM, TGA, XRD, and EDX analyses. This magnetic nanostructure, a new candidate for *in vitro* hyperthermia processes with a considerable saturation magnetization value (48.80 emu g^−1^) and heterogeneity, was exposed to an AMF. To evaluate the efficiency of the magnetic cross-linked Pec–Cel hydrogel nanobiocomposite, different concentrations of this new nanostructure (0.5 mg mL^−1^, 1.0 mg mL^−1^, 2.0 mg mL^−1^, 5.0 mg mL^−1^, and 10.0 mg mL^−1^) were examined at different frequencies (100.0 MHz, 200.0 MHz, 300.0 MHz, and 400.0 MHz), and the maximum specific absorption rate (126.0 W g^−1^) was attained by the least magnetic nanobiocomposite concentration (0.5 mg mL^−1^). Overall, it can be concluded that this designed magnetic nanobiocomposite can be considered for further investigation in hyperthermia therapy.

## Conflicts of interest

The authors listed in this article have no conflict of interest.

## Supplementary Material
